# Integrated metabolomics analysis identifies distinct amino acid signatures in chronic hepatitis B patients with metabolic dysfunction-associated steatotic liver disease

**DOI:** 10.3389/fcimb.2026.1783221

**Published:** 2026-04-29

**Authors:** Yanping Lan, Zitao Zhou, Qunfang Huang, Xin Yang, Xinrong Lu, Renquan Jiang, Yujue He, Xinyao Yang, Detai Ye, Zixin Chen, Can Liu, Qishui Ou, Zhen Xun

**Affiliations:** 1 Department of Laboratory Medicine, Fujian Key Laboratory of Laboratory Medicine, Gene Diagnosis Research Center, Fujian Clinical Research Center for Clinical Immunology Laboratory Test, The First Affiliated Hospital, Fujian Medical University, Fuzhou, Fujian, China; 2 Department of Laboratory Medicine, National Regional Medical Center, Binhai Campus of the First Affiliated Hospital, Fujian Medical University, Fuzhou, Fujian, China

**Keywords:** amino acid, biomarker, chronic hepatitis B, MASLD, metabolomics

## Abstract

**Background:**

Chronic hepatitis B (CHB) and metabolic dysfunction-associated steatotic liver disease (MASLD) frequently coexist and synergistically accelerate progression to end-stage liver disease. However, the metabolic differences distinguishing CHB alone from CHB with MASLD remain poorly defined.

**Methods:**

Serum samples from 47 CHB patients with or without MASLD were analyzed as a discovery set using widely targeted metabolomics. An independent validation set of 94 samples was subsequently examined with targeted metabolomics.

**Results:**

CHB patients with MASLD showed marked alterations in amino acid metabolism compared to those with CHB alone. Analysis revealed 206 significantly altered metabolites, among which amino acids represented the largest altered subgroup. Targeted quantification validated nine amino acids significantly upregulated in CHB-MASLD patients: leucine, tryptophan, phenylalanine, tyrosine, glutamic acid, alanine, histidine, lysine, and 2-aminoadipate. These 9 differential amino metabolites (9-DAM) robustly discriminated CHB with MASLD from CHB alone, achieving AUCs of 0.889 and 0.918 in the discovery and validation sets, respectively. The 9-DAM panel consistently outperformed controlled attenuation parameters (CAP) and correlated positively with CAP and liver stiffness measurements.

**Conclusions:**

CHB patients with MASLD exhibit a distinct disruption of circulating amino acid metabolism, reflecting unique metabolomic signatures. These findings highlight promising biomarkers and the pathophysiological significance of this comorbidity.

## 1 Introduction

Hepatitis B virus (HBV) infection remains a major global health burden, with approximately 290 million individuals living with chronic hepatitis B (CHB) worldwide (Cui et al., 2023; Lee et al., 2025). Persistent HBV infection can lead to progressive liver injury and severe end-stage liver diseases, including cirrhosis and hepatocellular carcinoma (HCC), representing a leading cause of liver-related morbidity and mortality (Zhuo et al., 2025). Meanwhile, the prevalence of metabolic dysfunction-associated steatotic liver disease (MASLD), formerly known as nonalcoholic fatty liver disease (NAFLD), has become the most prevalent chronic liver disease worldwide (Park et al., 2025; Smith et al., 2025). Accordingly, the coexistence of CHB and MASLD is increasingly recognized, with a systematic review estimating the prevalence of hepatic steatosis among CHB patients at approximately 30% (Zheng et al., 2021). This comorbidity exerts synergistic pathogenic effects, accelerating fibrosis progression and increasing the risk of cirrhosis and HCC (Lin et al., 2024; Kim et al., 2025). Such adverse outcomes may be driven by distinct molecular alterations in CHB-MASLD patients, yet the precise metabolic differences between CHB alone and CHB-MASLD remain unclear.

Accumulating evidence points to complex interactions between HBV infection and MASLD. On the one hand, hepatic steatosis appears to suppress HBV replication, as reflected by reduced serum HBV DNA levels and potentially increased clearance of HBV serological markers in CHB patients with concurrent MASLD (Huang et al., 2024). On the other hand, multiple studies demonstrate that hepatic steatosis accelerates fibrosis and heightens carcinogenic risk in CHB patients, although the mechanisms remain elusive (Kim et al., 2022). Advances in multi-omics have provided insights: transcriptomics revealed that steatosis-mediated suppression of interferon response pathways and related gene expression exacerbates fibrogenesis (Zhang et al., 2022); proteomics identified MASLD-associated protein expression linked to immune activation and metabolic dysregulation (Boel et al., 2025); and lipidomics highlighted lipoprotein-driven immune responses as central to early MASLD fibrosis progression (Lam et al., 2025). As MASLD is fundamentally a hepatic manifestation of systemic metabolic dysregulation, metabolic alterations likely underlie the unfavorable prognosis of CHB-MASLD comorbidity. However, comprehensive metabolomic analyses specifically addressing CHB-MASLD remain lacking.

Fibrosis severity is a key determinant of prognosis in CHB patients with steatosis, underscoring the need for accurate assessment (Rui et al., 2024a). Although liver biopsy remains the diagnostic gold standard, its invasiveness, sampling variability, and poor patient acceptance limit clinical utility. Noninvasive tests such as the Fibrosis-4 Index (FIB-4), Aspartate Aminotransferase to Platelet Ratio Index (APRI), and NAFLD Fibrosis Score (NFS) provide safer, more reproducible assessments (Pennisi et al., 2023). Nonetheless, their diagnostic accuracy in CHB-MASLD populations is insufficiently validated (Rui et al., 2024b). Meanwhile, metabolomics has become increasingly valuable in disease diagnosis and pathogenesis research (Tan et al., 2024). As a central tool in systems biology, it enables high-throughput qualitative and quantitative analysis of small-molecule metabolites using platforms such as high-resolution mass spectrometry (HRMS). However, its application to define the specific metabolic disorder and diagnostic potential in CHB-MASLD remains largely unexplored.

Therefore, we applied integrated, widely targeted, and targeted metabolomics to delineate the metabolic characteristics of CHB patients with hepatic steatosis and to characterize differences relative to patients with CHB alone. Our analyses revealed distinct alterations in amino acid metabolism and demonstrated significant correlations between specific amino acids and clinical indicators of steatosis and fibrosis, providing new metabolic insights into the progression of CHB-MASLD.

## 2 Materials and methods

### 2.1 Study population

Patients with CHB and concurrent MASLD diagnosed by ultrasonography at the First Affiliated Hospital of Fujian Medical University were enrolled. CHB was diagnosed by the persistence of hepatitis B surface antigen (HBsAg) or HBV DNA for more than 6 months. The diagnosis of MASLD was established according to the *Guideline for the Prevention and Treatment of Metabolic Dysfunction-associated Fatty Liver Disease (Version 2024)* (Fan et al., 2024), which requires fulfillment of two core criteria: (1) ultrasonographic evidence of hepatic steatosis, characterized by hepatorenal echo contrast, vessel blurring, and deep attenuation; and (2) the presence of at least one metabolic abnormality, including BMI ≥ 23 kg/m², elevated blood glucose, dyslipidemia, or elevated blood pressure. All CHB-MASLD patients met both diagnostic criteria, while CHB-only patients had no ultrasonographic evidence of hepatic steatosis.

The following exclusion criteria were applied: (1) infection with hepatitis C, D, or E virus, or human immunodeficiency virus; (2) other liver diseases such as alcohol-associated liver disease, autoimmune hepatitis, drug-induced steatosis, or other known chronic liver diseases; (3) presence of hepatocellular carcinoma or other malignancies; (4) history of decompensated cirrhosis, liver transplantation, or severe systemic diseases; (5) use of medications known to affect circulating metabolite levels, including statins, antidiabetic drugs, BCAA supplements, and vitamins.

The discovery and validation cohorts consisted of 47 and 94 CHB patients with or without MASLD, respectively (**Supplementary Tables S1**, **S2**). To further address the generalizability of our findings, an additional 30 patients with CHB-MASLD were subsequently enrolled for supplementary validation. The study adhered to the principles of the 1975 Declaration of Helsinki and was approved by the Ethics Committee of the First Affiliated Hospital of Fujian Medical University.

### 2.2 Laboratory measurements

Comprehensive clinical data, including demographic characteristics, medical history, and imaging findings, were collected from all participants. Complete blood cell counts were measured on a Siemens ADVIA 2120i automated hematology analyzer (Siemens Healthineers, Erlangen, Germany). Routine biochemical indicators, including ALT, AST, GGT, ALP, and other liver function markers, were determined using a Siemens Atellica CH 930 automated chemistry system (Siemens Healthcare Diagnostics, Tarrytown, USA). HBV DNA levels were quantified using the Roche Cobas TaqMan real-time PCR system (Roche Diagnostics, Basel, Switzerland). HBV serological markers (HBsAg, anti-HBs, HBeAg, anti-HBe) were assessed with an Abbott i2000SR automated chemiluminescence immunoassay system (Abbott Laboratories, Chicago, USA).

### 2.3 Widely targeted metabolomic profiling analysis

Metabolites were extracted by protein precipitation. Each fasting serum sample was mixed with a methanol-based extraction solution containing 20% acetonitrile and vigorously vortexed. Following centrifugation at 12,000 rpm for 10 minutes at 4 °C, the supernatant was transferred to a fresh tube and precipitated at –20 °C for 30 minutes. A second centrifugation at 12,000 rpm for 3 minutes at 4 °C ensured complete protein removal. All extracts were stored at -80 °C with a standardized time-to-freeze protocol. A 180 μL aliquot of the clarified supernatant was then analyzed by ultra-performance liquid chromatography (UPLC) coupled with tandem mass spectrometry (MS/MS) under optimized chromatographic and mass spectrometric conditions. The sample analysis order was randomized to mitigate batch effects.

A pooled quality control (QC) sample, prepared by combining equal aliquots of all extracts, was analyzed repeatedly (every 10 injections) using an LC-QTOF-MS/MS platform. System stability was confirmed by the high overlap of total ion chromatograms from sequential QC runs. A mixture of internal standards was used for normalization, with coefficients of variation (CVs) below 15% in QC samples. Over 85% of detected metabolites exhibited a CV < 0.5 across QCs and a consistently high inter-QC Pearson correlation further validated the reproducibility of the entire workflow. Metabolites with a detection rate <80% in QC or study samples were excluded.

Metabolite identification was based on multiple databases, with ion pairs and retention times matched against Metware’s proprietary target database to construct a study-specific metabolite library. Quantification was performed on all samples using a Q-Trap instrument in multiple reaction monitoring (MRM) mode. Mass spectrometry data were processed with Analyst 1.6.3 software. Extracted ion chromatographic peaks were integrated, and peak areas for identical metabolites across samples were normalized and corrected.

### 2.4 Targeted quantitative assay validation

Absolute quantification of 28 amino acids was performed by Calibra Diagnostic Company (Hangzhou, China). These 28 metabolites were selected because they represent a standardized, clinically accessible panel that is routinely measured in hospital laboratories using commercially available diagnostic kits. While untargeted metabolomics can identify thousands of metabolic features, many of these are present at trace levels, lack absolute quantification standards. In contrast, the selected 28 amino acids are highly abundant in circulation and can be stably and reproducibly quantified in routine clinical practice. They constitute a comprehensive clinical panel for evaluating systemic nitrogen homeostasis and hepatic metabolic function, including essential amino acids, branched-chain amino acids (BCAAs), aromatic amino acids (AAAs), and key intermediates of the urea cycle. Serum samples were thawed at 4 °C, mixed with methanol containing internal standards (IS), vortexed, and centrifuged at 14,000 rpm for 10 minutes at 4 °C. The supernatant was evaporated under nitrogen, and the residue was derivatized with 50 μL of acetyl chloride in alcohol at 60 °C for 30 minutes. The mixture was again dried under nitrogen, reconstituted in 100 μL of 2% methanol in water, vortexed, centrifuged at 14,000 rpm for 10 minutes at 4 °C, and the supernatant injected for analysis under optimized chromatographic and mass spectrometric conditions.

Separation was achieved on a Phenomenex C18 column. Quantitative analysis was performed on a CalQuant-S LC-MS/MS system (Calibra Scientific, China) coupled to a CalTarget-CT350 platform in MRM mode. Raw data were processed using MultiQuant™ software (version 3.0.3). Peak areas of analytes and IS were extracted, and analyte concentrations were calculated based on peak area ratios (analyte/IS) against calibration curves.

### 2.5 Statistical analysis

Continuous variables were expressed as mean ± standard deviation. Normally distributed variables were compared using Student’s *t*-test or ANOVA. Correlations were assessed using Spearman’s rank correlation coefficient. Statistical analyses and graph generation were conducted using R (version 4.1.1) and GraphPad Prism (version 10). A *p*-value < 0.05 was considered statistically significant.

## 3 Results

### 3.1 Patient cohort and study design

**Figure 1:**
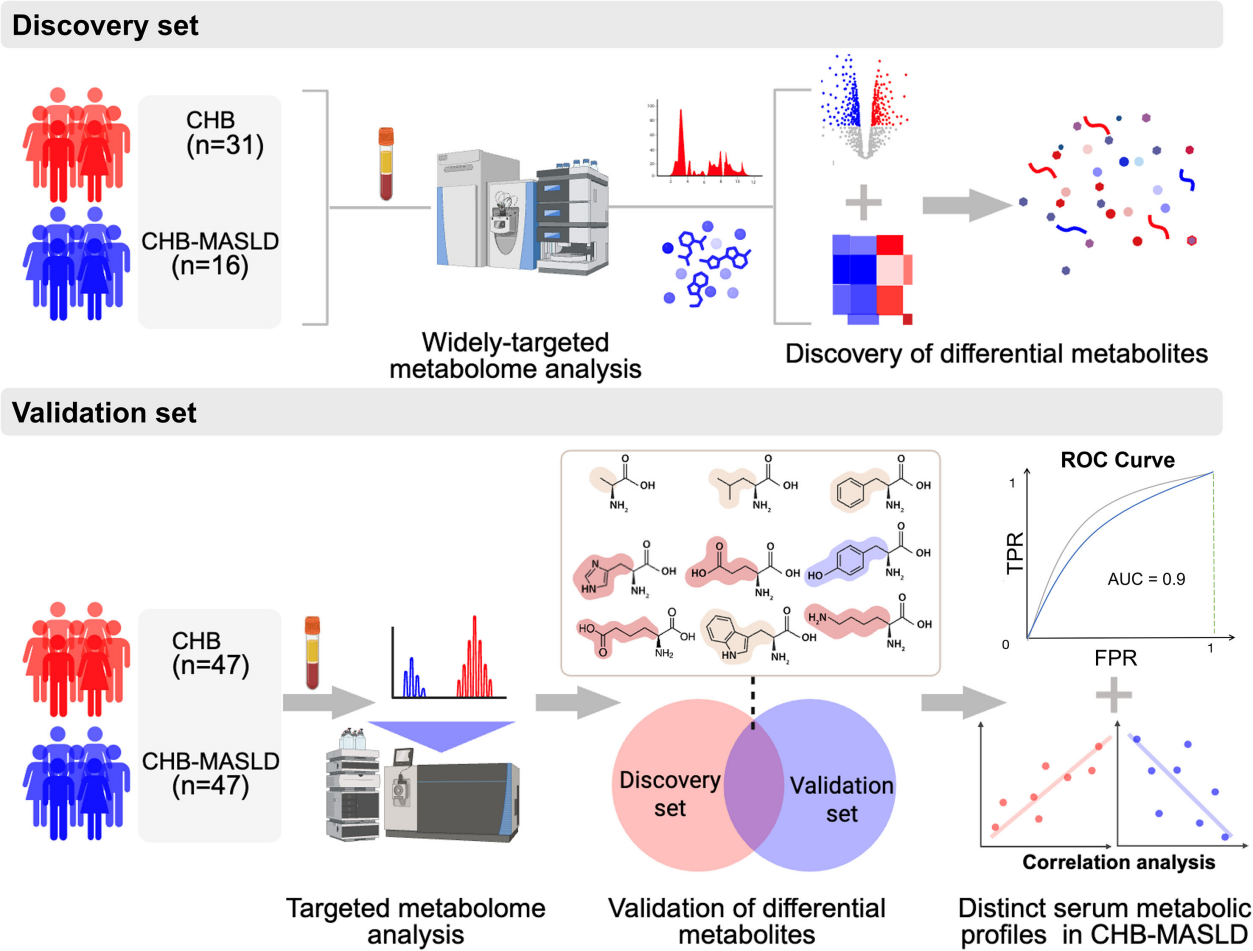
Workflow diagram of the study.

A total of 141 patients with chronic HBV infection were enrolled. The discovery cohort included 47 patients (16 with CHB-MASLD and 31 with CHB alone) (**Supplementary Table S1**), while the validation cohort comprised 94 patients (47 with CHB-MASLD and 47 with CHB alone) (**Supplementary Table S2**). Significant differences were observed in liver function indices and FibroScan measurements between the CHB-MASLD and the CHB groups. The overall workflow of the study is summarized in **Figure 1**. Widely targeted metabolomics was applied in the discovery set to identify differential metabolites, followed by bioinformatic analyses to screen candidate biomarkers. These candidates were subsequently validated in the independent cohort using targeted metabolomics, and their correlations with clinical parameters were further examined.

### 3.2 Altered serum metabolic profiles in CHB-MASLD patients

**Figure 2:**
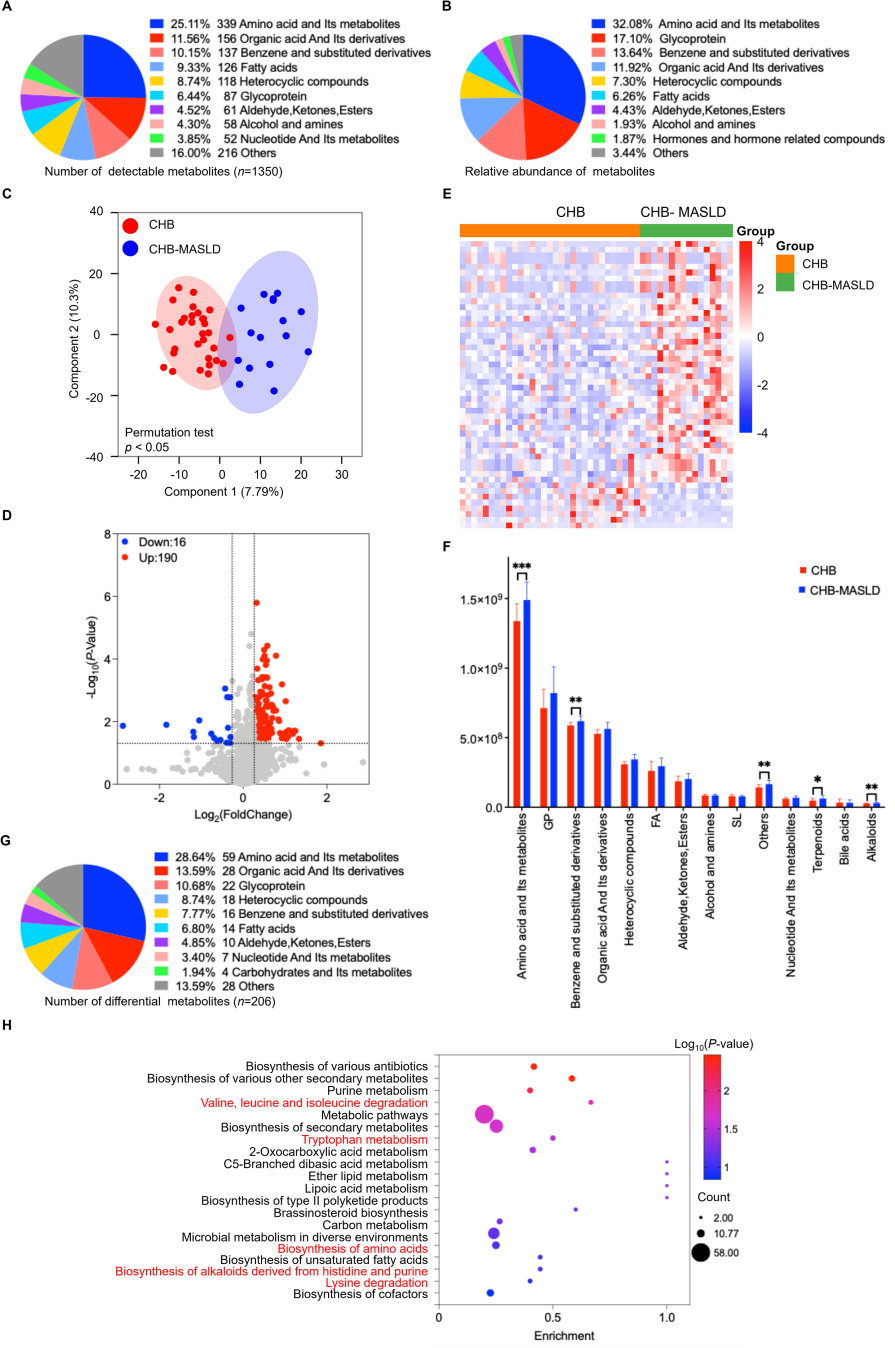
Diverse differential metabolites in CHB patients *vs*. CHB-MASLD patients in the discovery set. **(A)** Classification and quantity of detected serum metabolites (*n* = 1350). **(B)** Relative abundance of metabolites. **(C)** OPLS-DA score plot illustrating separation between CHB and CHB-MASLD groups. The OPLS-DA model was validated using a permutation test (n = 200) to prevent overfitting. **(D)** Volcano plot of differential metabolites. Significantly upregulated and downregulated metabolites are highlighted in red and blue. **(E)** Heatmap of 95 differential amino acid metabolites. Red denotes high expression and blue indicates low expression. **(F)** Bar plot showing differential metabolites in each subclass. Data were analyzed using an unpaired Student’s t-test and were presented as mean ± SD. *p < 0.05, ** *p* < 0.01, ***p < 0.001. **(G)** Proportion of differential metabolites in each subclass. **(H)** KEGG pathway enrichment of differential metabolites.

Untargeted metabolomics identified 1,350 serum metabolites with known structures, grouped into 10 subclasses (**Figure 2A**). Amino acid metabolites were the most numerous (*n* = 339), followed by organic acids and their derivatives (*n* = 156). Relative abundance analysis confirmed that amino acids were the dominant metabolite class (**Figure 2B**). OPLS-DA revealed a clear separation between CHB-MASLD and CHB patients (**Figure 2C**), indicating distinct metabolic signatures. Differential abundance analysis identified 206 metabolites (**Figures 2D, E**; **Supplementary Table S3**), with amino acids comprising the largest and most significant subgroup (**Figures 2F, G**). KEGG enrichment analysis demonstrated that these differential metabolites were primarily associated with amino acid metabolism pathways (**Figure 2H**), suggesting that amino acid dysregulation is a hallmark of CHB-MASLD patients.

### 3.3 Differential amino acid metabolites in CHB-MASLD patients in the discovery set

**Figure 3:**
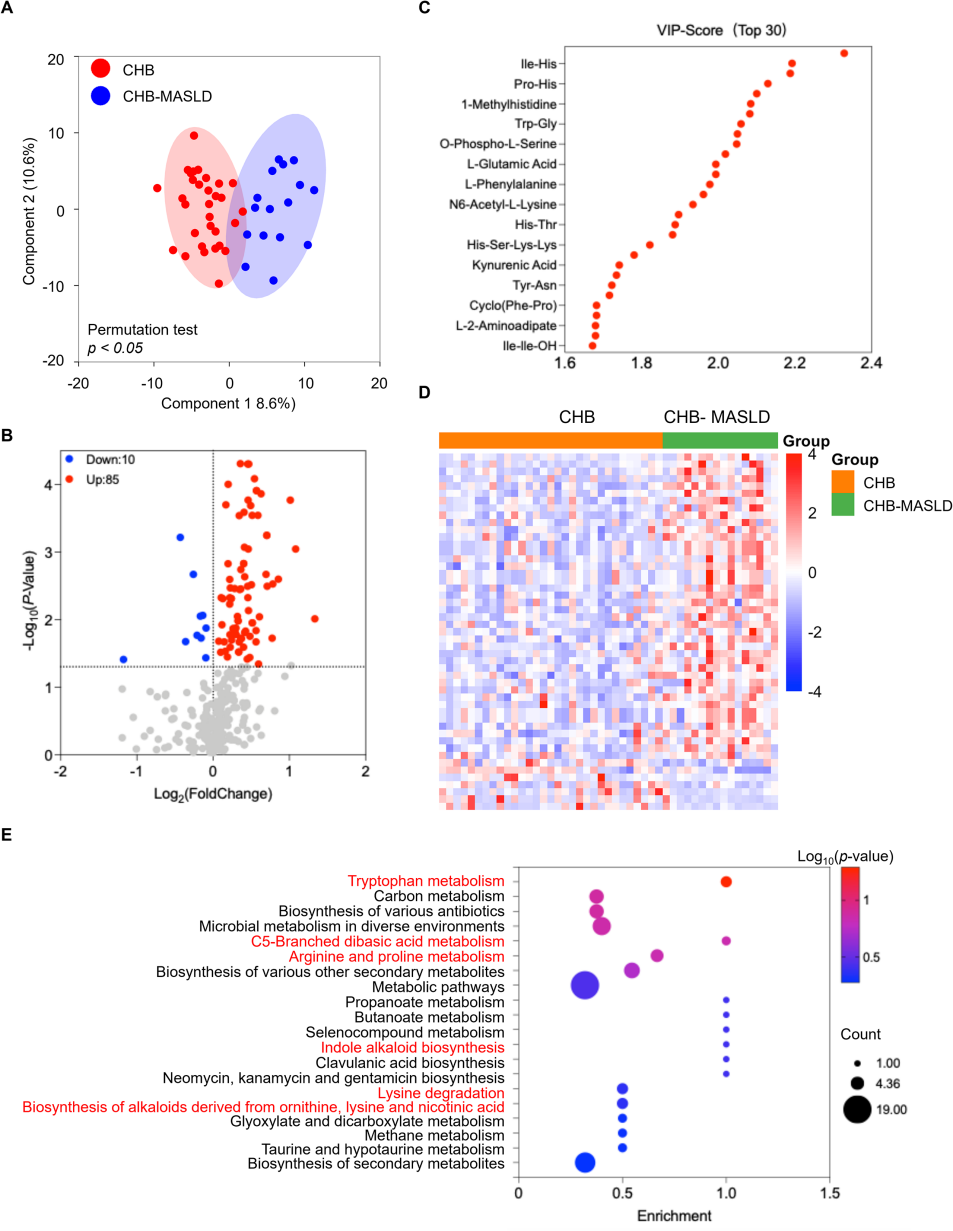
Differential amino acid metabolite analysis in CHB patients *vs*. CHB-MASLD patients in the discovery set. **(A)** OPLS-DA score plot from amino acid metabolic profiles. The OPLS-DA model was validated using a permutation test (n = 200) to prevent overfitting. **(B)** Volcano plot showing significantly altered amino acids (VIP>1, *p* < 0.05). **(C)** Variable importance in projection (VIP) scores of the top 30 metabolites contributing to group discrimination. **(D)** Heatmap of 95 differential amino acid metabolites. **(E)** KEGG pathway enrichment analysis of differentially expressed amino acids. Data were analyzed using Student’s t-test or Wilcoxon rank-sum test, depending on normality and variance homogeneity.

Amino acid profiling revealed striking metabolic distinctions between CHB-MASLD and CHB patients. OPLS-DA confirmed that amino acid signatures could robustly discriminate the two groups (**Figure 3A**). Combined univariate and multivariate analyses (VIP > 1, *p* < 0.05) identified 95 differential amino acid metabolites, including 85 upregulated and 10 downregulated in CHB-MASLD patients (**Figures 3B, C**; **Supplementary Table S4**). Notably, these downregulated metabolites primarily consist of seven oligopeptides (Arg-Ser, Gln-Lys-Phe-Arg, Gly-Val, Leu-Leu-Leu-Leu-Ser, Met-Asp, Met-Phe-Thr-Glu-Asp, and Tyr-Glu-Val-Lys) and three acetylated amino acids (N-Acetyl-L-Histidine, Nα-Acetyl-L-Arginine, and O-Acetyl-L-serine). Heatmap visualization highlighted clear intergroup differences (**Figure 3D**). KEGG enrichment analysis indicated significant involvement of tryptophan metabolism, branched-chain amino acid (BCAA) metabolism, lysine metabolism, and other amino acid-related pathways (**Figure 3E**), underscoring characteristic amino acid disturbances in CHB-MASLD patients.

### 3.4 Validation of amino acid metabolism differences in the validation set

**Figure 4:**
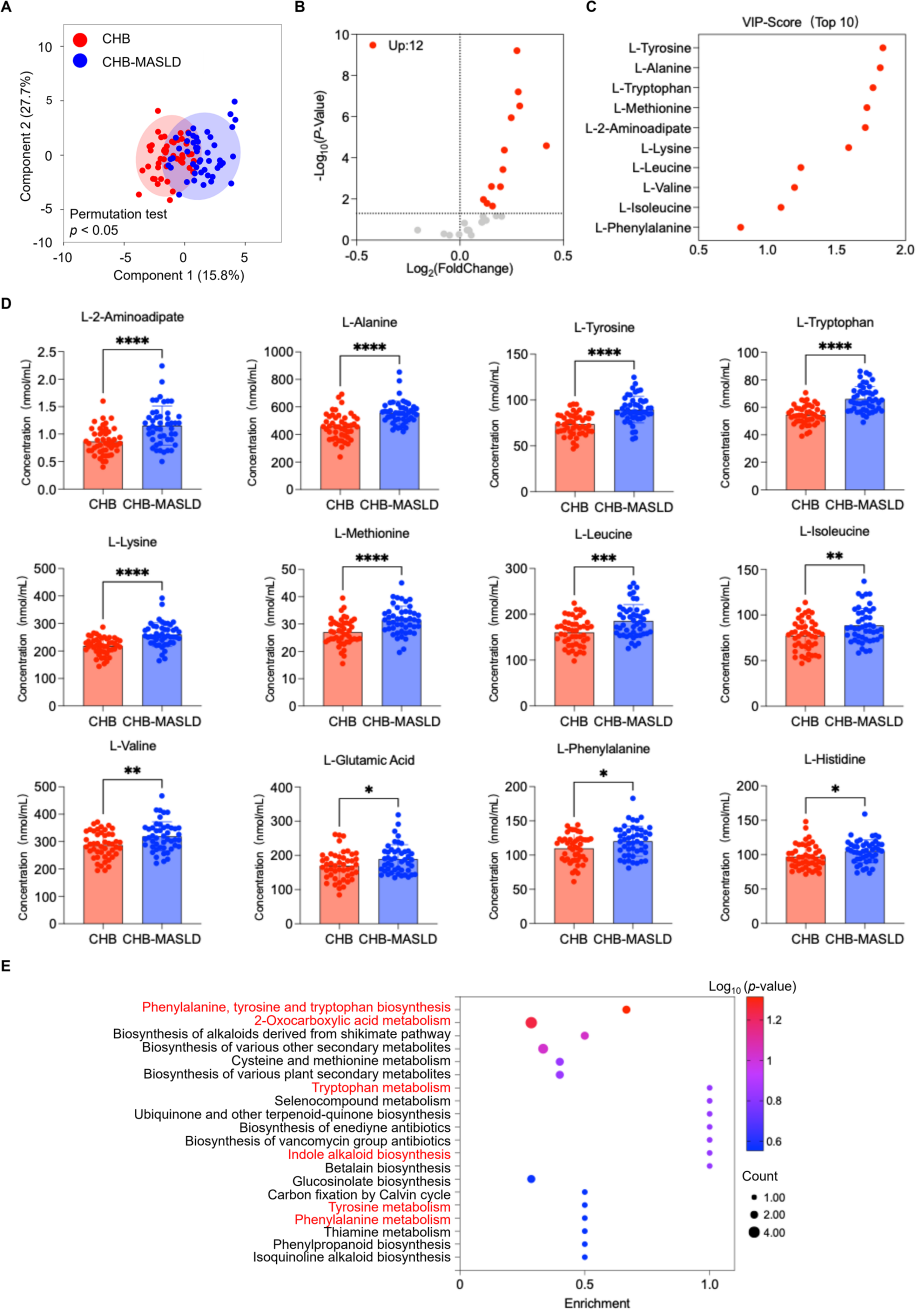
Validation of differential amino acid metabolites in CHB patients *vs*. CHB-MASLD patients in the validation set. **(A)** OPLS-DA score plot based on 28 targeted amino acids. The OPLS-DA model was validated using a permutation test (n = 200) to prevent overfitting. **(B)** Volcano plot of differential amino acids (*p* < 0.05). **(C)** Variable importance in projection (VIP) scores of the top 10 metabolites contributing to group discrimination. **(D)** Concentration comparisons of 12 significantly altered amino acids between groups. Data were analyzed using an unpaired Student’s *t*-test and were presented as mean ± SD. *p < 0.05, **p < 0.01, ***p < 0.001, ****p < 0.0001. **(E)** KEGG pathway enrichment analysis of 12 differentially expressed amino acids. Data were analyzed using Student’s t-test or Wilcoxon rank-sum test, depending on normality and variance homogeneity.

In the discovery set, we identified 95 differentially expressed amino acid metabolites using widely targeted metabolomics. To facilitate clinical applicability, we subsequently focused on 28 amino acids routinely quantified in clinical practice and validated them in 94 patients (47 CHB and 47 CHB-MASLD). OPLS-DA demonstrated group discrimination, though with partial overlap (**Figure 4A**). Using a threshold of *p* < 0.05, 12 amino acids showed significant intergroup differences: L-Methionine, L-Histidine, L-Glutamic acid, L-Isoleucine, L-Phenylalanine, L-Valine, L-Tyrosine, L-Lysine, L-Leucine, L-Tryptophan, L-Alanine, and L-2-Aminoadipic acid (**Figures 4B–D**; **Supplementary Table S5**). KEGG analysis again highlighted enrichment in amino acid metabolism pathways, including tryptophan, BCAA, and aromatic amino acid metabolism (**Figure 4E**). The consistent upregulation of these amino acids in the validation set reinforced the presence of dysregulated amino acid metabolism in CHB-MASLD patients.

### 3.5 Identification of amino acid biomarkers for discriminating MASLD in CHB patients

**Figure 5:**
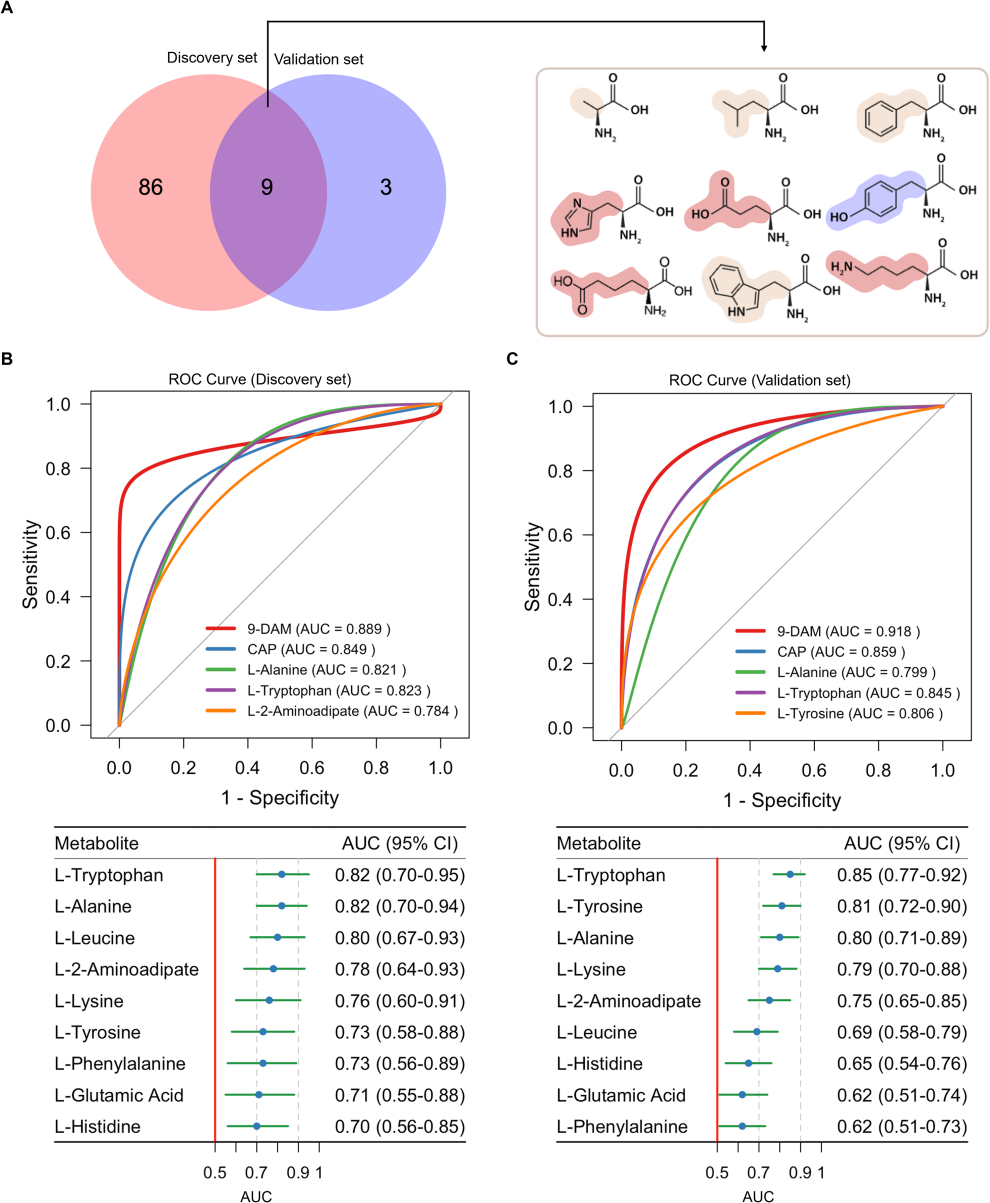
Discovery of amino acid metabolic biomarkers in CHB and CHB-MASLD patients. **(A)** Venn diagram showing overlap of differential amino acid metabolites between the discovery (95 metabolites) and validation (12 metabolites) sets, identifying 9 shared metabolites. **(B, C)** ROC curves of the 9-DAM panel and individual metabolites in the discovery **(B)** and validation **(C)** set. The 9-DAM panel consistently outperformed CAP in both cohorts. The 95% CI was calculated using the Delong test.


Integration of the 95 differential amino acids identified in the discovery set with the 12 amino acids confirmed in the validation set revealed 9 overlapping metabolites (**Figure 5A**). These metabolites included L-2-Aminoadipic acid, L-Alanine, L-Glutamic acid, L-Histidine, L-Leucine, L-Lysine, L-Phenylalanine, L-Tryptophan, and L-Tyrosine. A logistic regression model incorporating these nine amino acids was constructed to generate an integrated biomarker panel, termed the “9-DAM” (9 differential amino metabolites). The score can be calculated using the following equation:

The 9−DAM score =−22.031223 − 0.593078×L−2−Aminoadipic acid + 0.009937×L−Alanine + 0.012562×L−Glutamic acid − 0.057990×L−Histidine + 0.003858×L−Leucine + 0.028685×L−Lysine + 0.005269×L−Phenylalanine + 0.197697×L−Tryptophan + 0.015086×L−Tyrosine.

Receiver operating characteristic (ROC) curve analysis demonstrated robust diagnostic performance, with area under the curve (AUC) values of 0.889 (95% CI, 0.838-0.958) in the discovery set and 0.918 (95% CI, 0.863-0.972) in the validation set (**Figures 5B, C**). Importantly, the 9-DAM panel outperformed FibroScan-derived Controlled Attenuation Parameter (CAP) in both cohorts (**Figures 5B, C**), underscoring its potential as a non-invasive diagnostic tool for CHB-MASLD in clinical practice.

To mitigate the risk of overfitting arising from feature selection in the discovery set, we first assessed multicollinearity by calculating the Variance Inflation Factor (VIF) for the nine selected metabolites. All VIF values were below 10 (ranging from 1.55 to 8.94) (**Supplementary Table S6**), confirming that the features are independent predictors and the model is not inflated by redundant variables. Furthermore, we added a 5-fold cross-validation within the targeted quantification cohort, which yielded a consistent and high diagnostic performance (average AUC = 0.857) (**Supplementary Figures S1A, B**), demonstrating the internal stability of our feature selection process. This was supplemented by a leave-one-out cross-validation (LOOCV) within the same cohort, which yielded a robust AUC of 0.853, thereby demonstrating the internal stability of the finalized clinical model (**Supplementary Figure S1C**). Finally, bootstrap resampling (1000 iterations) yielded an optimism-corrected AUC of 0.873, confirming that the model’s predictive power was not driven by overfitting (**Supplementary Figure S1D**).

To evaluate the robustness of the 9-DAM panel, we conducted a multivariable analysis in the validation set that adjusted for age, sex, BMI, glucose, ALT, HBV DNA, HBeAg status, total protein, urea, GGT, and eGFR. The panel remained a strong and independent indicator of CHB−MASLD, with an adjusted odds ratio of 3.94 (95% CI, 2.13–10.23) (**Supplementary Table S7**). Notably, BMI and ALT showed statistical significance in analyses confined to clinical variables, but did not retain significance after the 9−DAM panel was included. This finding implies that the metabolic profile reflected by the 9−DAM panel incorporates the key pathophysiological information represented by these conventional indicators. Furthermore, the addition of the panel markedly improved the ability to distinguish between the groups, raising the AUC from 0.843 to 0.939 (*p* < 0.05) (**Supplementary Table S7**). Furthermore, we conducted a comprehensive comparative analysis between the 9-DAM panel and several widely used clinical scores, including FIB-4, APRI, and AAR. Our results demonstrate that the 9-DAM panel exhibits significantly superior diagnostic performance, achieving an AUC of 0.889 in the discovery set and 0.918 in the validation set. In contrast, conventional scores showed limited discriminative power in this specific CHB-MASLD context (Discovery: APRI AUC = 0.601, AAR AUC = 0.590, FIB-4 AUC = 0.490; validation: APRI AUC = 0.620, AAR AUC = 0.648, FIB-4 AUC = 0.565) (**Supplementary Figures S2A, B**).

We additionally performed a multivariable logistic regression analysis incorporating both the 9-DAM signature and CAP values to determine their independent diagnostic contributions. In the discovery set, the 9-DAM signature remained a robust independent predictor of CHB-MASLD (*p* = 0.003), whereas CAP failed to reach statistical significance within the same model (*p* = 0.081). This superiority was further confirmed in the validation set, although both parameters reached statistical significance (*p* < 0.001), the 9-DAM score exhibited a substantially higher odds ratio compared to CAP (1.79 vs. 1.04), highlighting its primary diagnostic weight. Notably, the combination of the 9-DAM score and CAP yielded higher AUC values (discovery: 0.927, validation: 0.949). This improvement was statistically significant compared to CAP alone (*p* = 0.042), yet no significant difference was found between the combination and the 9-DAM panel individually (**Supplementary Table S8**). Ultimately, integrating the 9-DAM panel with CAP maximized the overall diagnostic efficacy, achieving the highest AUC values of 0.927 and 0.949 in the discovery and validation sets, respectively (**Supplementary Figures S2A, B**). Together, the 9-DAM panel reflects the core metabolic reprogramming of the liver, providing a higher diagnostic weight and more granular pathological insight than current standard-of-care physical or biochemical markers.

### 3.6 Correlation of differential amino acid biomarkers with hepatic steatosis and fibrosis in CHB and CHB-MASLD patients

**Figure 6:**
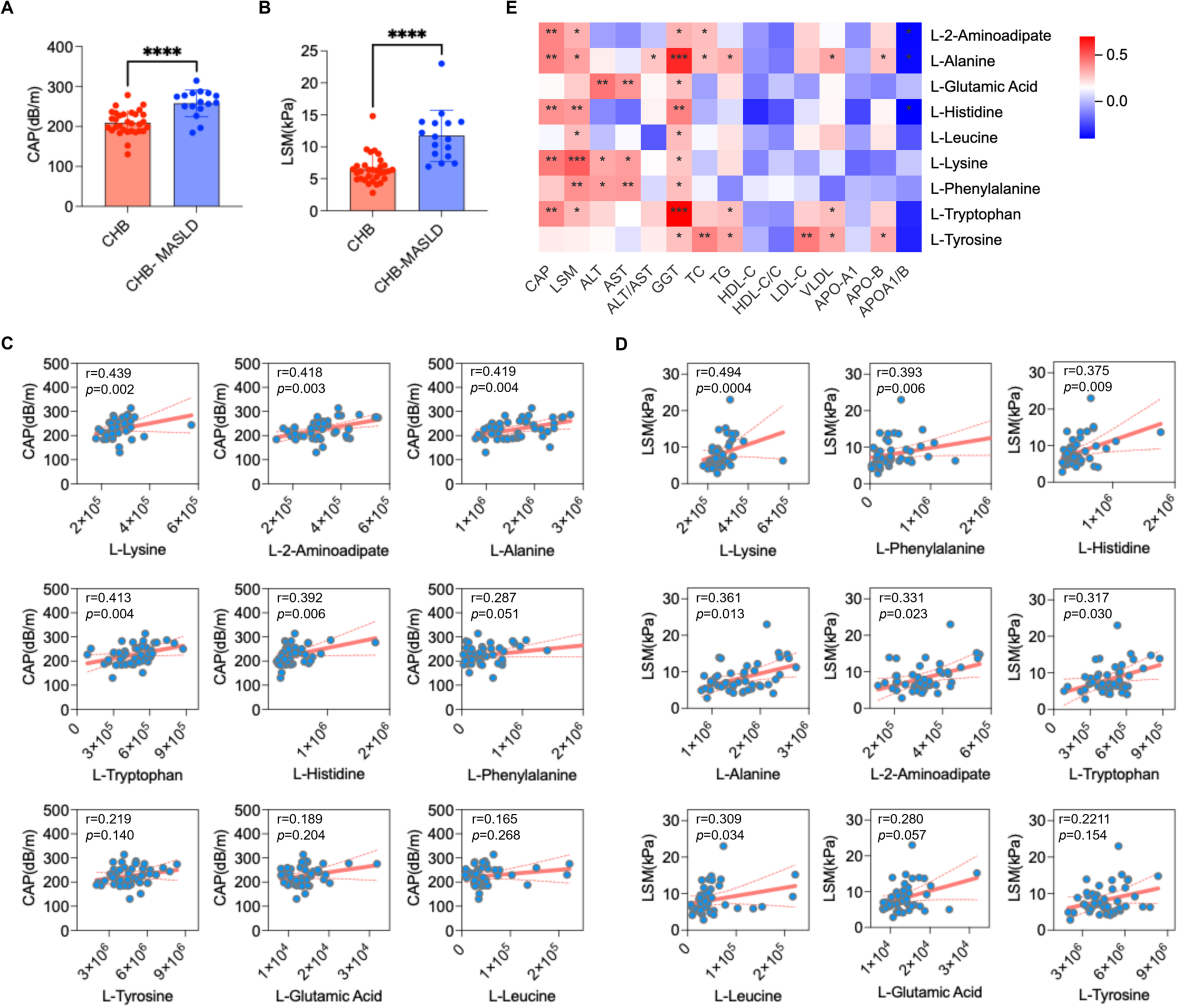
Correlation of amino acid biomarkers with hepatic steatosis, fibrosis, and clinical indices in CHB and CHB-MASLD patients. **(A)** Comparison of CAP between CHB and CHB-MASLD groups. **(B)** Comparison of LSM between CHB and CHB-MASLD groups. **(C)** Correlation of the nine amino acid metabolites with CAP. **(D)** Correlation of the nine amino acid metabolites with LSM. **(E)** Heatmap showing associations between the nine amino acid metabolites and liver function markers (GGT, ALT, AST) and lipid profiles (TC, TG, LDL-C). Data in **(A, B)** were analyzed using an unpaired Student’s *t*-test and are presented as mean ± SD. ****p < 0.0001. Correlation analyses in **(C–E)** were performed using Spearman’s rank correlation, with r values indicating correlation coefficients. *p < 0.05, **p < 0.01, ***p < 0.001.

To investigate the relationship between these nine amino acid biomarkers and hepatic injury in CHB-MASLD, we compared FibroScan-derived parameters between groups. CAP and liver stiffness measurement (LSM) values were significantly higher in CHB-MASLD than in CHB patients, reflecting greater hepatic steatosis and fibrosis (**Figures 6A, B**). Correlation analyses showed that five of the nine metabolites were positively associated with CAP (**Figure 6C**), while seven correlated positively with LSM (**Figure 6D**), further supporting their link with disease severity. In addition, several metabolites displayed positive associations with liver function indices (GGT, ALT, AST) and lipid parameters (TC, TG, LDL-C) (**Figure 6E**). Collectively, these findings highlight a strong connection between amino acid metabolic dysregulation and the clinical manifestations of steatosis, fibrosis, and metabolic dysfunction in CHB-MASLD, implicating these metabolites in the pathogenesis of this comorbid condition.

To rigorously distinguish metabolic shifts driven by steatosis from those secondary to fibrosis, we have added a new subgroup analysis evaluating the 9-DAM panel in a subgroup of patients with minimal fibrosis (F0–F1, LSM < 7.4 kPa). Notably, the panel maintained robust diagnostic accuracy for CHB-MASLD in this subgroup, achieving an AUC of 0.794 in the discovery cohort and 0.944 in the validation cohort (**Supplementary Figures S3A, B**). These additional results demonstrate that the signature is not merely a reflection of advanced liver fibrosis. Furthermore, we performed an additional validation using an independent cohort of 30 patients with MASLD alone (HBV-negative) to confirm the MASLD-specific nature of these markers. Our comparative analysis revealed that seven of the nine key metabolites—L-2-Aminoadipate, L-Lysine, L-Tyrosine, L-Alanine, L-Glutamic Acid, L-Phenylalanine, and L-Histidine—showed no significant differences between the MASLD-only and CHB-MASLD groups (**Supplementary Figure S3C**). Although L-Tryptophan and L-Leucine exhibited significant differences between the two groups, which may reflect a synergistic viral-metabolic interaction or altered proteolysis unique to the CHB-MASLD milieu, the overall stability of the majority of the panel reinforces its specificity to the MASLD component. we further evaluated the integrated 9-DAM score across these cohorts. Notably, the overall 9-DAM score showed no significant difference between the MASLD-only and CHB-MASLD groups, while both remained significantly higher than the CHB group (**Supplementary Figure S3D**). This high degree of consistency indicates that these metabolic alterations are primarily driven by the underlying metabolic dysfunction associated with MASLD, rather than being exclusive to CHB infection or fibrosis severity.

## 4 Discussion

This study investigated the metabolomic profiles of CHB patients with and without MASLD, compared molecular characteristics between groups, and evaluated the utility of widely targeted and targeted metabolomic approaches for diagnosis. The major findings are: (a) nine differential amino acid metabolites were identified that effectively discriminate CHB-MASLD from CHB patients with high sensitivity and specificity; (b) CHB-MASLD patients exhibited prominent disruptions in amino acid metabolism, with these metabolites enriched in amino acid–related pathways; and (c) these metabolites correlated significantly with hepatic steatosis (CAP) and fibrosis severity (LSM), supporting their potential as noninvasive diagnostic biomarkers; (d) and these metabolites exhibited a consistent elevation pattern in an independent cohort of patients with MASLD alone, suggesting that this 9-DAM signature is closely associated with hepatic steatosis, regardless of the HBV infection status.

From a diagnostic perspective, existing noninvasive approaches for assessing liver fibrosis in CHB-MASLD are constrained by limited sensitivity and specificity, often yielding inconclusive results (Rui et al., 2024a). Recently developed noninvasive tests (NITs) have not adequately considered the impact of MASLD on the metabolic profile of CHB patients, reducing diagnostic accuracy in this subgroup. By contrast, our metabolomics-based approach effectively distinguished CHB-MASLD from CHB and MASLD by capturing characteristic amino acid metabolic signatures, highlighting its promise as a novel diagnostic strategy. Moreover, the targeted measurement of these nine amino acids is cost-effective and amenable to high-throughput clinical testing, enhancing its potential for practical implementation.

Mechanistically, amino acids are critical carbon sources for hepatic lipid synthesis and play essential roles in MASLD pathogenesis (Liao et al., 2024). Our findings suggest that the 9-DAM signature reflects three interconnected pathways of hepatic dysregulation driven by the concurrent presence of MASLD and CHB: Firstly, the elevation of aromatic amino acids (Phe, Tyr, Trp) and leucine reflects a decline in hepatic metabolic clearance and proteostasis. While chronic liver disease typically reduces circulating BCAAs due to ammonia detoxification demands, their elevation in CHB-MASLD–particularly leucine–suggests a deleterious cycle. Elevated BCAAs can exacerbate insulin resistance and persistently activate the mTOR signaling pathway, which supports protein synthesis and promotes cancer cell proliferation, linking this signature directly to HCC progression (Mahendran et al., 2017; Wang et al., 2025). Secondly, alterations in alanine, glutamic acid, and histidine signify a redirection of nitrogen scavenging. This shift suggests an increased anaplerotic flux into the TCA cycle, likely a compensatory response to combat lipid-induced oxidative stress. Finally, the lysine and 2-aminoadipate (2-AAA) axis serves as a mechanistic marker for mitochondrial remodeling and early-stage insulin resistance within the CHB-MASLD milieu. Furthermore, the collective downregulation of these ten additional metabolites suggests a broader alteration in hepatic metabolic homeostasis. Although excluded from the final panel due to higher variability, their depletion likely points to altered protein turnover and shifts in amino acid acetylation processes, providing additional context to the complex metabolic reprogramming observed in CHB-MASLD. Collectively, these findings suggest that MASLD acts as a “metabolic accelerator”, transforming chronic inflammatory stress into a state of systemic metabolic exhaustion where the liver’s homeostatic capacity is fundamentally compromised.

Aromatic amino acids (AAAs: tyrosine, tryptophan, phenylalanine), metabolized primarily in the liver, also play crucial roles in disease progression. The Fisher ratio (AAA/BCAA) is widely used to evaluate liver function and the risk of hepatic encephalopathy (HE) (Soeters and Fischer, 1976). Lipid accumulation impairs AAA metabolism, and tryptophan, in particular, closely interacts with the gut microbiome. While its metabolite indole-3-acetic acid improves insulin sensitivity and reduces oxidative stress (Zhao et al., 2019), aberrant tryptophan metabolism activates the aryl hydrocarbon receptor (AhR) pathway, driving liver carcinogenesis (Chen et al., 2022). Similarly, tyrosine and phenylalanine are markedly elevated in liver disease (Morgan et al., 1978; Chen et al., 2022). Phenylalanine is converted to tyrosine by phenylalanine hydroxylase, while phenylalanyl-tRNA synthetase (FARS) can catalyze covalent modification of insulin receptor β (IRβ) by phenylalanine, suppressing IRβ kinase activity, blocking insulin signaling, and inducing systemic insulin resistance (Zhou et al., 2022). In CHB-MASLD patients, impaired AAA metabolism leads to their accumulation, aggravating disease progression through multiple mechanisms: phenylalanine–IRβ modification promotes insulin resistance and lipid dysregulation; aberrant tryptophan metabolism activates AhR signaling, driving inflammation, fibrosis, and malignant transformation; and AAA–BCAA imbalance impairs ammonia detoxification, elevating HE risk. Thus, AAA accumulation represents a prominent metabolic signature closely associated with metabolic dysfunction, insulin resistance, fibrosis, and carcinogenesis in CHB-MASLD. While the cross-sectional nature of this study precludes definitive causal inference, these findings highlight a complex interplay between amino acid dysregulation and disease progression.

Other amino acids also contributed to disease progression. Glutamic acid, a central intermediate in amino acid transamination and TCA cycle entry, was significantly elevated. Prior studies reported downregulation of hepatic enzymes involved in glutamate metabolism in MASH, with glutamate accumulation largely driven by γ-glutamyl transferase–mediated release during glutathione turnover, closely associated with fibrosis (Hasegawa et al., 2020). Alanine, a key mediator of the liver–islet axis, influences glucagon secretion, and the glucagon–alanine index correlates strongly with insulin resistance and steatosis severity (Winther-Sørensen et al., 2020). Elevated histidine may be converted to histamine, which perturbs lipid transport and fatty acid uptake via AMPK and v-ATPase activation, exacerbating steatosis, inflammation, and fibrosis (Deng et al., 2024). Increased lysine promotes *de novo* fatty acid synthesis, hepatocyte ballooning, and inflammation, while its metabolite 2-aminoadipate—also elevated in our study—impairs lipid metabolism, reduces HDL-C, and increases insulin levels, accelerating MASLD progression (Tuomola et al., 2025). Collectively, these metabolic perturbations parallel the severity of hepatic inflammation and fibrosis, potentially reflecting or exacerbating the pathophysiological shifts that lead to unfavorable long-term prognoses in CHB-MASLD patients. Further longitudinal studies are warranted to delineate whether these metabolic changes are drivers or consequences of advancing liver disease.

This study has several limitations. First, notwithstanding the potential of noninvasive diagnosis, integrating traditional and novel indicators using artificial intelligence-based machine learning models could improve the prediction of histological changes and disease progression in CHB-MASLD. Second, our findings do not elucidate the mechanistic basis of the observed metabolic alterations; the analysis was lacked pathway-level integration with key signaling cascades that may drive disease progression. Finally, despite the lack of liver tissue validation or experimental models to definitively link circulating metabolites to hepatic pathology, the 9-DAM panel’s strong correlation with liver-specific parameters (CAP and LSM) underscores its hepatic relevance. These metabolic signatures likely mirror the liver’s impaired metabolic capacity, aligning with established histological evidence (Zhao et al., 2019; Liao et al., 2024; Cheng et al., 2025). To move beyond correlation, future studies utilizing multi-omics, animal models, or hepatic tissue analysis are essential to establish causation and elucidate the underlying pathogenic mechanisms.

In conclusion, this study demonstrates distinctive disruptions of amino acid metabolism in CHB-MASLD, enabling differentiation from CHB alone. These findings highlight amino acid dysregulation as a key contributor to disease progression and unfavorable prognosis and support the development of amino acid–based biomarkers as noninvasive tools for diagnosis and risk stratification in CHB-MASLD.

## Data Availability

The original contributions presented in the study are included in the article/**Supplementary Material**. Further inquiries can be directed to the corresponding authors.
