# Supplementary material for: Integrated metabolomics analysis identifies distinct amino acid signatures in chronic hepatitis B patients with metabolic dysfunction-associated steatotic liver disease
Source: Front Cell Infect Microbiol. 2026 Apr 29;16:1783221. doi: 10.3389/fcimb.2026.1783221 (PMC13167716; doi:10.3389/fcimb.2026.1783221)
Supplement: Supplementary file 1 [file DataSheet1.pdf]

Table S1. Population characteristics in the discovery set.

Characteristics	CHB (*n*=31)	CHB-MASLD (*n*=16)	*P*-Value
Age (years)	35.90 (9.59)	35.50 (7.10)	0.883
CAP (dB/m)	209.32 (30.06)	257.50 (33.51)	<0.001
LSM (kPa)	6.91 (2.54)	10.88 (4.65)	<0.001
TBIL (μmol/L)	13.39 (5.98)	15.44 (9.83)	0.380
DBIL (μmol/L)	4.22 (1.66)	4.66 (2.08)	0.441
IBIL (μmol/L)	9.17 (4.46)	10.78 (7.89)	0.375
Total Protein (g/L)	72.12 (4.26)	75.11 (4.25)	0.027
ALB (g/L)	45.14 (3.07)	45.91 (3.17)	0.428
GLB (g/L)	26.98 (3.77)	29.21 (4.09)	0.069
ALB/GLB	1.71 (0.31)	1.60 (0.28)	0.255
ALT (U/L)	48.58 (27.57)	60.81 (21.09)	0.128
AST (U/L)	34.58 (17.91)	41.19 (16.53)	0.225
ALT/AST	1.39 (0.39)	1.52 (0.22)	0.240
GGT (U/L)	24.42 (15.65)	41.31 (21.94)	0.004
LDH (U/L)	180.65 (31.80)	200.44 (34.93)	0.057
ALP (U/L)	72.00 (22.77)	76.25 (21.61)	0.541
UREA (mmol/L)	4.42 (1.00)	5.02 (0.86)	0.046
CREA (μmol/L)	70.40 (13.34)	70.25 (22.32)	0.977
UA (μmol/L)	336.34 (67.95)	380.38 (133.98)	0.140
eGFR	94.44 (18.60)	108.0 (17.24)	0.019
GLU (mmol/L)	4.96 (0.61)	4.98 (0.70)	0.923
TC (mmol/L)	4.84 (1.19)	5.12 (1.23)	0.459
TG (mmol/L)	1.11 (0.36)	1.34 (0.72)	0.149
HDL-C (mmol/L)	1.47 (0.39)	1.30 (0.34)	0.148
HDL-C/TC	0.31 (0.08)	0.28 (0.10)	0.420
TG/HDL-C	0.84 (0.45)	1.14 (0.79)	0.100
LDL-C (mmol/L)	3.16 (1.10)	3.52 (1.24)	0.308
VLDL (mmol/L)	0.51 (0.16)	0.59 (0.31)	0.254
APO-A1 (g/L)	1.48 (0.27)	1.33 (0.23)	0.068
APO-B (g/L)	0.95 (0.29)	1.02 (0.34)	0.492
APOA1/B	1.65 (0.45)	1.42 (0.45)	0.099
HBsAg (log_{10} IU/ml)	4.18 (0.70)	4.09 (0.59)	0.659
HBeAg (log_{10} IU/ml)	2.41 (1.18)	2.76 (0.61)	0.271
HBV-DNA (log_{10} IU/ml)	7.15 (1.30)	7.79 (0.68)	0.070
AFP (ng/mL)	3.95 (2.03)	3.98 (1.85)	0.966

Variables are expressed as the mean (standard) for continuous data, and as number (percentage) for categorical data. CAP, controlled attenuation parameter; LSM, liver stiffness measurement; TBIL, total bilirubin; DBIL, direct bilirubin; IBIL, indirect bilirubin; ALB, albumin; GLB, globulin; A/G, albumin-to-globulin ratio; ALT, alanine aminotransferase; AST, aspartate aminotransferase; GGT, gamma-glutamyl transferase; LDH, lactate dehydrogenase; ALP,

alkaline phosphatase; CREA, creatinine; UA, uric acid; GLU, glucose; TC, total cholesterol; TG, triglycerides; HDL-C, high-density lipoprotein cholesterol; LDL-C, low-density lipoprotein cholesterol; VLDL, very low-density lipoprotein; APO-A1, apolipoprotein A1; APO-B, apolipoprotein B; HBsAg, hepatitis B surface antigen; HBeAg, hepatitis B e antigen; HBV-DNA, hepatitis B virus deoxyribonucleic acid; AFP, alpha-fetoprotein.

Table S2. Population characteristics in the validation set.

Characteristics	CHB (*n*=47)	CHB-MASLD (*n*=47)	*P*-Value
Age (years)	38.83 (8.10)	42.34 (10.61)	0.075
CAP (dB/m)	213.85 (34.38)	266.98 (36.12)	<0.001
LSM (kPa)	5.28 (1.79)	6.15 (1.84)	0.022
TBIL (μmol/L)	13.02 (5.00)	13.97 (5.30)	0.376
DBIL (μmol/L)	3.67 (1.79)	3.66 (1.73)	0.977
IBIL (μmol/L)	9.35 (3.42)	10.33 (3.90)	0.198
Total Protein (g/L)	74.09 (3.71)	76.56 (4.81)	0.006
ALB (g/L)	45.80 (2.36)	47.21 (2.91)	0.012
GLB (g/L)	28.31 (3.83)	29.29 (3.79)	0.216
ALB/GLB (A/G)	1.64 (0.31)	1.64 (0.24)	0.891
ALT (U/L)	23.98 (11.11)	36.45 (28.83)	0.007
AST (U/L)	24.40 (7.88)	31.55 (15.28)	0.005
ALT/AST	0.98 (0.31)	1.15 (0.33)	0.012
GGT (U/L)	17.64 (9.69)	28.96 (21.69)	0.002
LDH (U/L)	187.87 (37.19)	215.68 (48.65)	0.002
ALP (U/L)	64.11 (20.40)	72.64 (16.02)	0.027
UREA (mmol/L)	4.69 (0.99)	4.88 (1.01)	0.353
CREA (μmol/L)	69.57 (15.46)	77.11 (12.86)	0.012
UA (μmol/L)	315.17 (74.37)	357.47 (90.21)	0.015
eGFR	99.06 (19.10)	93.11 (14.56)	0.093
GLU (mmol/L)	4.99 (0.68)	5.44 (0.95)	0.009
TC (mmol/L)	4.78 (0.84)	5.21 (1.05)	0.029
TG (mmol/L)	1.16 (0.50)	1.39 (0.51)	0.031
HDL-C (mmol/L)	1.51 (0.45)	1.28 (0.29)	0.004
HDL-C/TC	0.32 (0.09)	0.27 (0.09)	0.005
TG/HDL-C	0.89 (0.59)	1.17 (0.56)	0.025
LDL-C (mmol/L)	3.06 (0.75)	3.59 (1.01)	0.005
VLDL (mmol/L)	0.53 (0.23)	0.65 (0.25)	0.014
APO-A1 (g/L)	1.55 (0.33)	1.47 (0.24)	0.171
APO-B (g/L)	0.92 (0.20)	1.10 (0.28)	0.001
APOA1/B	1.78 (0.55)	1.53 (0.98)	0.129
HBsAg (log_{10} U/ml)	3.54 (1.02)	3.38 (0.87)	0.429
HBeAg (log_{10} U/ml)	0.79 (1.54)	0.66 (1.49)	0.693
HBV-DNA (log_{10} U/ml)	4.99 (2.25)	4.63 (2.23)	0.431
AFP (ng/mL)	2.49 (1.14)	2.74 (1.22)	0.292

Variables are expressed as the mean (standard) for continuous data, and as number (percentage) for categorical data. CAP, controlled attenuation parameter; LSM, liver stiffness measurement; TBIL, total bilirubin; DBIL, direct bilirubin; IBIL, indirect bilirubin; ALB, albumin; GLB, globulin; A/G, albumin-to-globulin ratio; ALT, alanine aminotransferase; AST, aspartate aminotransferase; GGT, gamma-glutamyl transferase; LDH, lactate dehydrogenase; ALP, alkaline phosphatase; CREA, creatinine; UA, uric acid; GLU, glucose; TC, total cholesterol; TG, triglycerides; HDL-C, high-density lipoprotein cholesterol; LDL-C, low-density

lipoprotein cholesterol; VLDL, very low-density lipoprotein; APO-A1, apolipoprotein A1; APO-B, apolipoprotein B; HBsAg, hepatitis B surface antigen; HBeAg, hepatitis B e antigen; HBV-DNA, hepatitis B virus deoxyribonucleic acid; AFP, alpha-fetoprotein.

Table S3. 206 differential metabolites in the discovery set.

Compounds	*P*-Value	Type	Log_2FC
(+/-)-Pavine	0.017	Up	0.317
(3beta,5alpha,6beta,7alpha,22E,24R)-Ergosta-8,22-diene-3,5,6,7-tetrol	0.009	Up	0.541
(R)-2-Hydroxybutyric acid	0.024	Up	0.567
(S)-3-Methyl-2-oxopentanoic acid	<0.001	Up	0.564
(S)-Leucic acid	0.002	Up	0.548
(Z)-2-tetracos-15-enamidoethanesulfonic acid	0.006	Up	0.303
(Z)-7-Hexadecen-1,16-olide	0.021	Up	0.457
[2-Hydroxy-1-(4-methoxyphenyl)propyl] 4-methoxybenzoate	0.024	Up	1.185
1,4-Dihydro-1-Methyl-4-Oxo-3-Pyridinecarboxamide	0.008	Up	0.468
12alpha-Hydroxy-3-oxochola-4,6-dienoate	0.003	Up	0.278
13(R)-HODE	0.022	Up	0.658
1-Aminocyclohexanecarboxylic acid	0.025	Up	0.301
1-Methyladenosine	0.038	Up	0.507
1-Methylhistidine	0.001	Up	0.587
2-(4-Hydroxyphenyl)ethanol	0.020	Up	1.227
2,3-Dinor fluprostenol	0.028	Up	0.532
2,4-Dihydroxy Benzoic Acid	0.034	Up	0.922
2,4-Dihydroxypteridine	0.006	Up	0.396
20,26-dihydroxyecdysone	0.005	Up	0.286
2-azaniumylethyl [(2R)-2-hydroxy-3-octadecanoyloxypropyl] phosphate	0.037	Up	0.340
2-Hydroxy-2-Methyl Butyric acid	0.025	Up	0.492
2-Hydroxy-3-Methyl Butanoic Acid	0.019	Up	0.506
2-Hydroxyhexanoic acid	0.002	Up	0.548
2-Hydroxyisocaproic Acid	0.002	Up	0.548
2-keto-D-gluconic acid	0.049	Up	0.364
2-Methyl-1-Pyrroline	<0.001	Up	0.321
2-Methyl-3-hydroxybutyric acid	0.002	Up	0.335
2-Stearoylglycerophosphoinositol	0.003	Up	0.352
3-[1-(3-Aminopropyl)-1H-indol-3-yl]-4-(1-methyl-1H-indol-3-yl)-1H-pyrrole-2,5-dione	0.013	Up	0.395
3alpha-Acetomethoxy-11alpha-oxo-12-ursen-24-oic acid	0.017	Up	0.452
3-Amino-2-piperidinone	0.008	Up	0.402
3-Amino-4-Hydroxybenzoic Acid	0.039	Up	0.326
3-Amino-5-hydroxybenzoic acid	0.039	Up	0.326
3-Dehydroteasterone	0.039	Up	0.493
3-Hydroxy-3-Methyl Butyric Acid	0.002	Up	0.323
3-Hydroxyanthranilic Acid	0.039	Up	0.326
3-Hydroxy-L-phenylalanine	0.011	Up	0.364

4-acetoxyphenol	0.011	Up	0.444
4-Hydroxybenzoic Acid	0.020	Up	1.245
4-Hydroxybenzyl alcohol	0.049	Up	0.323
4-Methyl-2-oxovaleric acid	0.003	Up	0.372
4-Pyridoxic Acid	0.049	Up	0.525
4-tert-butylbenzoic acid	0.002	Down	-0.380
5(Z),14(Z)-Eicosadienoic Acid	0.019	Up	0.470
5,6-DiHETrE	0.003	Up	0.651
5,7-Dihydroxy-2-phenyl-6-[3,4,5-trihydroxy-6-(hydroxymethyl)oxan-2-yl]-8-(3,4,5-trihydroxyoxan-2-yl)chromen-4-one	0.013	Up	0.365
5-Aminoimidazole ribonucleotide	0.011	Up	0.643
5-Aminovaleric Acid	0.017	Up	0.426
5-Hydroxyhexanoic Acid	0.002	Up	0.548
5-Hydroxyisourate	0.003	Up	0.688
6alpha-Hydroxy-castasterone	0.036	Up	0.332
7-Oxateasterone	0.003	Up	0.547
AA	<0.001	Up	0.448
Aminomalonic Acid	0.014	Up	0.273
Ansatrienin A	0.041	Up	0.452
Arg-Ser-Lys-Arg	0.019	Up	0.414
Asn-Phe-Ala-Arg	0.010	Up	0.503
Bufalin	0.009	Up	0.841
C-8 Ceramide-1-phosphate	0.009	Up	0.508
Caffeine	0.050	Up	1.854
Carnitine C11:1	0.043	Up	0.525
Carnitine C4:DC	0.018	Up	0.478
Carnitine C5:0	0.003	Up	0.588
Carnitine-2-methyl-C4	0.003	Up	0.588
Choline	0.009	Up	0.277
Cinegalline	0.002	Down	-0.315
Cyclo(Ala-Pro)	0.004	Up	0.453
cyclo(gly-glu)	0.010	Up	0.856
Cyclo(Phe-Pro)	0.032	Up	0.513
Cyclo(Pro-Phe)	0.032	Up	0.513
Cyclo(Pro-Val)	0.026	Up	0.480
Cys-Glu-Arg	0.028	Up	0.399
D-(+)-sucrose	0.017	Up	0.635
Decarboxy-Norlobaric Acid	0.016	Up	0.380
Dehydrofalcarinone	0.006	Up	0.369
D-Glucoronic Acid	0.035	Up	0.322
DHA	0.042	Up	0.421
Diethyl sebacate	0.034	Down	-0.697
Diisononyl phthalate	0.007	Up	0.542

DL-2-hydroxystearic acid	0.003	Up	0.317
DL-Stachydrine	0.024	Down	-0.762
D-Ornithine	0.008	Up	0.362
Dutasteride	0.001	Up	0.926
Eucalyptone	0.045	Up	0.470
Fasciculic acid A	0.049	Down	-0.320
FFA(18:3)	0.006	Up	0.531
FFA(20:4)	<0.001	Up	0.449
FFA(22:4)	0.041	Up	0.358
Furosemide	0.007	Up	0.285
Gibberellin	0.027	Up	0.313
Gln-Arg	0.022	Up	0.283
Glu-Leu	<0.001	Up	0.492
Glu-Thr-His-Glu	0.036	Up	1.335
Glu-Val	<0.001	Up	0.542
Gly-Val	0.031	Down	-1.176
Heterodendrin	0.025	Up	0.404
His-Ser-Lys-Lys	0.001	Up	0.629
His-Thr	0.019	Up	1.083
Hydroxyurea	0.035	Up	0.266
Ile-Gly-Ser	0.025	Up	0.464
Ile-His	<0.001	Up	0.784
Ile-Met	0.001	Up	0.513
Indole-3-acetamide	0.007	Up	0.359
Inosine	0.014	Down	-2.872
Isolithocholic acid	0.012	Up	0.378
Jurubine	0.014	Up	0.292
Kinamycin D	<0.001	Up	0.335
Kynurenic Acid	0.008	Up	0.406
L-2-Aminoadipate	0.006	Up	0.402
L-Alanine	0.044	Up	0.335
L-Allothreonine	0.039	Down	-0.543
L-Arginine	0.012	Up	0.299
Leu-Ala-Asn-Phe-Lys	0.011	Up	0.414
Leu-Leu-Leu-Leu-Ser	0.016	Down	-0.361
L-Glutamic Acid	<0.001	Up	0.570
L-Homoarginine	<0.001	Up	0.577
L-kynurenine	0.010	Up	0.350
L-Ornithine	0.014	Up	0.320
LPA(0:0/16:0)	0.005	Up	0.720
LPA(0:0/18:0)	0.034	Up	0.601
LPA(16:0/0:0)	0.017	Up	0.672
LPC(0:0/15:0)	0.044	Up	0.339
LPC(0:0/18:0)	0.018	Up	0.367

LPC(0:0/20:3)	0.028	Up	0.331
LPC(0:0/22:5)	0.016	Up	0.422
LPC(15:0/0:0)	0.044	Up	0.339
LPC(16:2/0:0)	0.024	Up	0.309
LPC(18:0/0:0)	0.018	Up	0.367
LPC(20:3/0:0)	0.028	Up	0.331
LPC(22:5/0:0)	0.008	Up	0.458
LPC(22:6/0:0)	0.029	Up	0.471
LPC(O-0:0/18:0)	0.050	Up	0.540
LPC(O-16:0/2:0)	0.018	Up	0.367
LPC(O-16:1/0:0)	0.023	Up	0.552
LPC(O-18:0/0:0)	0.050	Up	0.540
LPG(18:1/0:0)	0.035	Up	0.342
L-threo-3-Methylaspartate	<0.001	Up	0.570
L-Tryptophan	0.042	Up	0.342
Macrocarpal I	0.014	Up	0.590
Mandelic Acid	0.031	Up	1.070
Meliantriol	0.022	Up	0.624
Methylcysteine	0.001	Up	0.454
Methylmalonic Acid	0.026	Up	0.495
Momordol	0.036	Up	0.527
Moxifloxacin	0.002	Up	0.283
N(Alpha)-Acetyl-Epsilon-(2-Propenal)Lysine	0.002	Up	1.013
N-[2-(3,4-dimethoxyphenyl)ethyl]-3-(2-hydroxy-3,4-dimethoxyphenyl)prop-2-enimidic acid	0.017	Up	0.368
N6-(2-Hydroxyethyl)adenosine	0.009	Up	0.377
N6-Acetyl-L-Lysine	0.001	Up	0.411
N6-methyladenosine	0.038	Up	0.507
N-Acetylglucosamine 1-Phosphate	0.006	Up	0.351
N-Acetylhistamine	0.007	Up	0.455
N-Acetyl-L-Leucine	0.003	Up	0.343
N-Acetyl-L-phenylalanine	0.014	Up	0.288
N-Acetyl-L-Tyrosine	0.010	Up	0.456
N-Acetylneuraminic Acid(SA)	0.021	Down	-1.192
N-acetylpyrrolidine	<0.001	Up	0.502
N-lactoyl-phenylalanine	0.027	Up	0.563
N-Methyl-L-Glutamate	0.006	Up	0.402
N-MethyTrans-4-Hydroxy-Proline	0.009	Down	-1.048
O-Acetyl-L-serine	0.001	Down	-0.432
Octoxinol	0.018	Up	0.380
Oleoylglycerone phosphate	0.023	Up	0.686
O-Phospho-L-Serine	<0.001	Up	0.358
Pantothenate	0.005	Up	0.354
PC(O-16:0/0:0)	0.027	Up	0.620

PC(O-16:0/O-1:0)	0.006	Up	0.298
PC(O-16:0/O-2:0)	0.050	Up	0.540
Phe-Met	0.014	Up	0.372
Phe-Pro	0.041	Up	0.361
Phe-Ser	0.041	Down	-0.626
P–Hydroxyphenyl Acetic Acid	0.031	Up	1.070
Pinolenic acid	0.047	Up	0.382
Pinoresinol	0.013	Up	0.581
Primaquine	0.050	Up	0.565
Pristimerin	0.036	Up	1.030
Pro-Asn	<0.001	Up	0.468
Pro-His	0.004	Up	0.782
Pro-Met	0.005	Up	0.461
Rosiglitazone	0.013	Up	0.329
Sericetin	0.002	Up	0.390
Sn-Glycero-3-Phosphocholine	0.003	Up	0.676
Sodium cholate	0.013	Down	-1.834
Sphingosyl-phosphocholine	0.049	Up	0.562
Succinic Acid	0.026	Up	0.495
Taprostene	0.010	Up	0.417
Terephthalic Acid	0.001	Up	0.665
Testosterone	0.023	Up	0.879
Testosterone cypionate	0.026	Up	0.940
Tetracycline	0.021	Up	1.184
Tetrahca	0.049	Up	0.466
Thiamine	0.048	Down	-0.393
Thr-Leu-Lys-Lys	0.003	Up	0.709
Thr-Tyr-Arg-Lys	0.013	Up	0.608
Thr-Val-Leu-Thr-Ser	0.008	Up	0.695
Tri(Beta-Chloroethyl) Phosphate	0.031	Down	-0.305
TriacetonaMine	0.004	Up	0.354
Trp-Gly	<0.001	Up	0.508
Tumonoic Acid I	0.007	Up	0.337
Turmeronol B	0.022	Up	0.312
Tyr-Asn	0.001	Up	0.459
Uric acid	0.022	Up	1.016
Vindoline	0.007	Up	0.394
Xanthine	0.016	Up	0.415
Xanthosine	0.038	Up	0.295
β-Alanine	0.044	Up	0.335

Table S4. 95 differential amino acid metabolites in the discovery set.

Compounds	*P*-Value	Type	Log_2FC
1-Methylhistidine	<0.001	Up	0.587
2-Methylhippuric acid	0.018	Up	0.298
3-Hydroxy-L-phenylalanine	0.002	Up	0.364
5-Aminovaleric Acid	0.016	Up	0.426
6-Aminocaproic Acid	0.005	Up	0.238
Acetylvaline	0.031	Up	0.161
Ala-Lys	0.011	Up	0.327
Arg-Ser	0.037	Down	-0.096
Arg-Ser-Lys-Arg	0.017	Up	0.414
Asn-Phe-Ala-Arg	0.003	Up	0.503
Cyclo(Ala-Pro)	0.003	Up	0.453
cyclo(glu-glu)	0.002	Up	0.196
cyclo(gly-glu)	0.003	Up	0.856
Cyclo(Phe-Pro)	0.012	Up	0.513
Cyclo(Pro-Phe)	0.012	Up	0.513
Cyclo(Pro-Val)	0.039	Up	0.480
Cycloleucine	0.027	Up	0.224
Cys-Glu-Arg	0.027	Up	0.399
D-Allo-Isoleucine	0.004	Up	0.225
DL-Leucine	0.005	Up	0.238
D-Ornithine	0.004	Up	0.362
D-Phenylalanine	<0.001	Up	0.167
Gln-Arg	0.004	Up	0.283
Gln-Lys-Phe-Arg	0.013	Down	-0.092
Glu-Leu	<0.001	Up	0.492
Glu-Met	0.023	Up	0.562
Glu-Thr-His-Glu	0.010	Up	1.335
Glu-Val	<0.001	Up	0.542
Gly-Val	0.039	Down	-1.176
His-Ser-Lys-Lys	<0.001	Up	0.629
His-Thr	0.001	Up	1.083
Ile-Gly-Ser	0.008	Up	0.464
Ile-His	<0.001	Up	0.784
Ile-lle-OH	0.005	Up	0.107
Ile-Met	<0.001	Up	0.513
Ile-Thr	0.001	Up	0.705
Kynurenic Acid	<0.001	Up	0.406
L-2-Aminoadipate	0.002	Up	0.402
L-Alanine	0.032	Up	0.335
L-Arginine	0.014	Up	0.299
Leu-Ala-Asn-Phe-Lys	0.002	Up	0.414
Leu-Leu-Leu-Leu-Ser	0.021	Down	-0.361

Leu-Thr	0.001	Up	0.705
L-Glutamic Acid	<0.001	Up	0.570
L-Histidine	0.013	Up	0.115
L-Homoarginine	<0.001	Up	0.577
L-kynurenine	0.004	Up	0.350
L-Lysine	0.006	Up	0.217
L-Ornithine	0.010	Up	0.320
L-Phenylalanine	<0.001	Up	0.198
L-threo-3-Methylaspartate	<0.001	Up	0.570
L-Tryptophan	0.023	Up	0.342
L-Tyrosine	0.018	Up	0.221
Lys-Ala	0.011	Up	0.327
Lys-Ala-Leu-Glu	0.019	Up	0.482
Lys-Ser	0.023	Up	0.145
Met-Asp	0.002	Down	-0.259
Met-Glu	0.041	Up	0.448
Methylcysteine	<0.001	Up	0.454
Met-Phe-Thr-Glu-Asp	0.017	Down	-0.210
N(Alpha)-Acetyl-Epsilon-(2-Propenal)Lysine	<0.001	Up	1.013
N6-Acetyl-L-Lysine	0.001	Up	0.411
N-Acetyl-L-Histidine	0.009	Down	-0.168
N-Acetyl-L-Leucine	<0.001	Up	0.343
N-Acetyl-L-phenylalanine	0.017	Up	0.288
N-Acetyl-L-Tyrosine	0.005	Up	0.456
N-acetylornithine	0.038	Up	0.188
N-Ethylglycine	0.015	Up	0.262
N-Formylglycine	0.022	Up	0.242
N-lactoyl-phenylalanine	0.016	Up	0.563
N-Methylalanine	0.015	Up	0.262
N-Methyl-L-Glutamate	0.002	Up	0.402
N-Phenylacetylglycine	0.018	Up	0.298
N-Propionylglycine	0.005	Up	0.219
Nα-Acetyl-L-Arginine	0.009	Down	-0.135
O-Acetyl-L-serine	0.001	Down	-0.432
O-Phospho-L-Serine	<0.001	Up	0.358
Orlistat	0.003	Up	0.218
Phe-Met	0.021	Up	0.372
Phe-Pro	0.020	Up	0.361
Pro-Asn	<0.001	Up	0.468
Pro-His	0.003	Up	0.782
Pro-Ile	0.049	Up	0.596
Pro-Met	0.001	Up	0.461
Ser-Leu	0.020	Up	0.774

Ser-Nap-OH	0.005	Up	0.127
Thr-Leu-Lys-Lys	0.003	Up	0.709
Thr-Tyr-Arg-Lys	0.010	Up	0.608
Thr-Val-Leu-Thr-Ser	0.002	Up	0.695
Trp-Gly	<0.001	Up	0.508
Trp-Nap-OH	0.033	Up	0.100
Tyr-Asn	<0.001	Up	0.459
Tyr-Cys-Trp	0.022	Up	0.074
Tyr-Glu-Val-Lys	0.019	Down	-0.157
β-Alanine	0.032	Up	0.335

Table S5. Targeted quantification of 28 amino acids in the validation set.

Compounds	*P*-Value	Type	Log_2FC
Tryptophan	<0.001	Up	0.276
Tyrosine	<0.001	Up	0.282
Alanine	<0.001	Up	0.289
Lysine	<0.001	Up	0.248
2-Aminoadipate	<0.001	Up	0.418
Methionine	<0.001	Up	0.215
Leucine	<0.001	Up	0.208
Valine	0.003	Up	0.152
Isoleucine	0.003	Up	0.196
Histidine	0.012	Up	0.114
Phenylalanine	0.018	Up	0.131
Glutamic Acid	0.025	Up	0.157
1-Methyl-L-histidine	0.506	Insignificant	0.042
3-Methyl-L-histidine	0.511	Insignificant	-0.205
4-Hydroxyproline	0.907	Insignificant	0.054
α-Aminobutyric acid	0.208	Insignificant	0.107
Arginine	0.213	Insignificant	0.106
Aspartate	0.509	Insignificant	0.034
β-Aminoisobutyric acid	0.780	Insignificant	-0.077
Citrulline	0.910	Insignificant	-0.050
Glycine	0.827	Insignificant	-0.004
Ornithine	0.104	Insignificant	0.175
Proline	0.111	Insignificant	0.112
Sarcosine	0.111	Insignificant	0.202
Serine	0.233	Insignificant	0.022
Taurine	0.172	Insignificant	0.127
Threonine	0.562	Insignificant	0.045
β-Alanine	0.182	Insignificant	0.113

Table S6. Variance Inflation Factor (VIF) analysis of the nine candidate metabolites in the discovery set.

Metabolite	VIF
L-Alanine	8.939983
L-Tryptophan	5.607274
L-2-Aminoadipate	2.257881
L-Histidine	2.200541
L-Tyrosine	2.153388
L-Phenylalanine	1.808343
L-Glutamic Acid	1.784001
L-Leucine	1.677933
L-Lysine	1.553852

Table S7. Multivariate logistic regression analysis of factors associated with MASLD in CHB patients.

Variable	Clinical		Clinical+9-DAM	
	**aOR (95% CI)**	***P*-value**	**aOR (95% CI)**	***P*-value**
**Clinical Variables**				
Age (years)	1.06 (1.00-1.13)	0.062	1.05 (0.97-1.13)	0.242
Male sex	3.09 (0.99-10.41)	0.056	1.50 (0.32-7.29)	0.602
BMI (kg/m²)	1.23 (1.06-1.49)	0.021	1.15 (0.97-1.39)	0.116
Glucose (mmol/L)	1.71 (0.80-3.80)	0.173	1.70 (0.63-4.63)	0.291
ALT (U/L)	1.08 (1.02-1.14)	0.007	1.05 (0.99-1.14)	0.153
HBV DNA (log10 IU/mL)	1.06 (0.68-1.60)	0.798	1.14 (0.69-1.89)	0.607
HBeAg positive	0.82 (0.12-6.72)	0.841	0.34 (0.03-3.71)	0.374
Total Protein (g/L)	1.14 (1.00-1.32)	0.07	1.42 (1.12-1.93)	0.01
Urea (mmol/L)	0.90 (0.48-1.60)	0.721	0.51 (0.19-1.18)	0.133
GGT (U/L)	1.04 (1.00-1.10)	0.054	1.01 (0.96-1.08)	0.748
eGFR (mL/min/1.73m²)	0.99 (0.96-1.02)	0.467	1.02 (0.97-1.07)	0.497
**9-DAM Score**				
9-DAM Score	-	-	3.94 (2.13-10.23)	<0.001
**Performance**				
AUC	0.845		0.960	
95% CI	(0.763-0.922)		(0.926-0.995)	
Delong test	0.0008			

Data are presented as adjusted odds ratios (aOR) with 95% confidence intervals (95% CI).

Table S8. Multivariable logistic regression analysis of the 9-DAM panel and CAP for diagnosing CHB-MASLD.

	Discovery set		Validation set	
	aOR (95% CI)	P-value	aOR (95% CI)	*P*-value
**Variable**				
9-DAM score	1.88 (1.25-2.83)	0.003	1.79 (1.39-2.30)	<0.001
CAP (dB/m)	1.03 (1.00-1.07)	0.081	1.04 (1.02-1.06)	<0.001
**Performance of the Combined Model**				
AUC	0.927		0.949	
95% CI	(0.812-1)		(0.908-0.991)	
**Delong test**				
Combined vs. 9-DAM score	0.311		0.116	
Combined vs. CAP	0.133		0.042	

Data are presented as adjusted odds ratios (aOR) with 95% confidence intervals (95% CI).

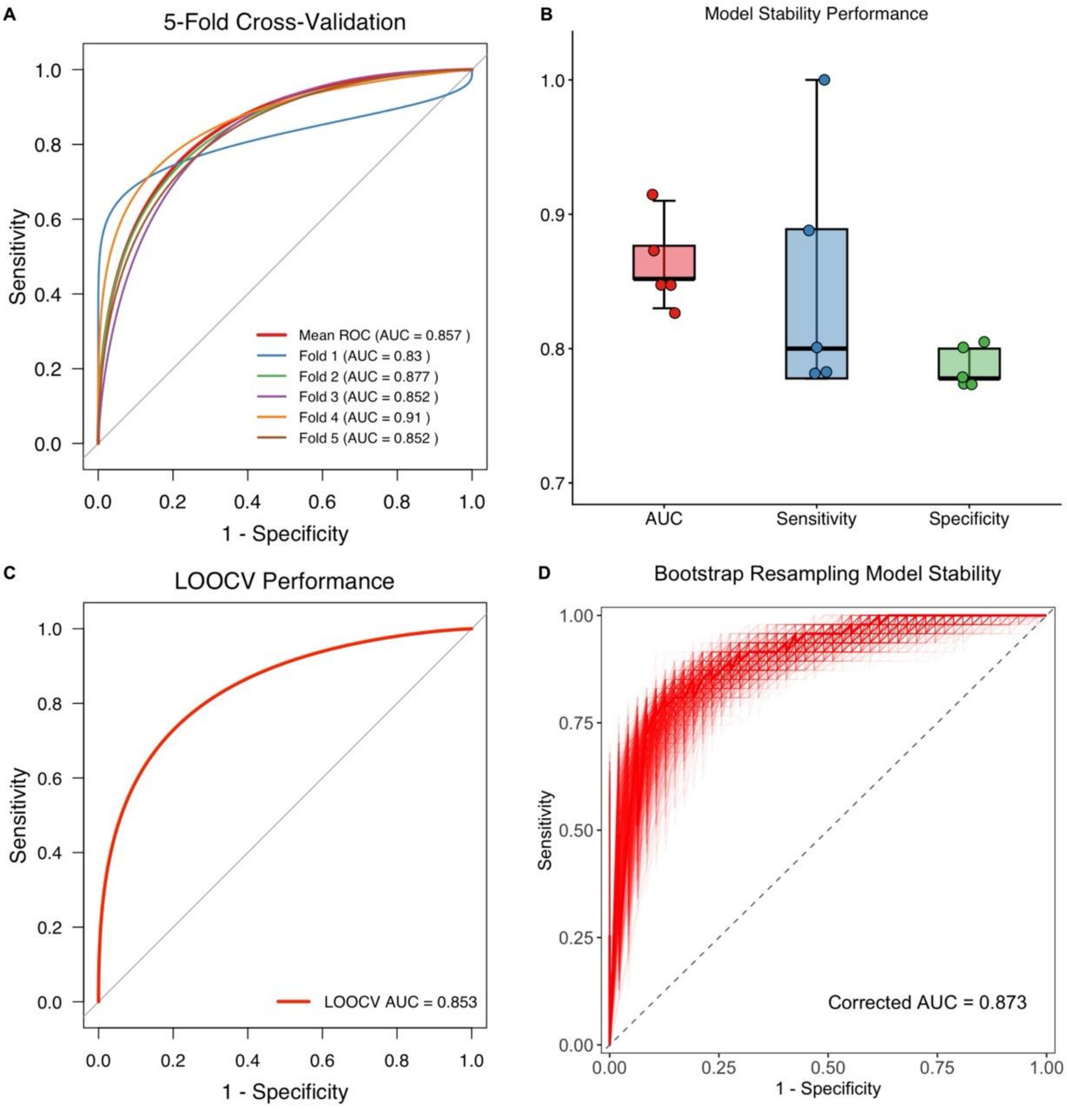

Fig. S1 Internal validation demonstrates the robustness of the 9-DAM panel. (**A-B**) ROC curves and stability performance of the 9-DAM panel across 5-fold cross-validation. (**C**) Performance evaluation of the 9-DAM panel using leave-one-out cross-validation (LOOCV). (**D**) Bootstrap resampling traces (1000 iterations) showing the derivation of the optimism-corrected AUC.

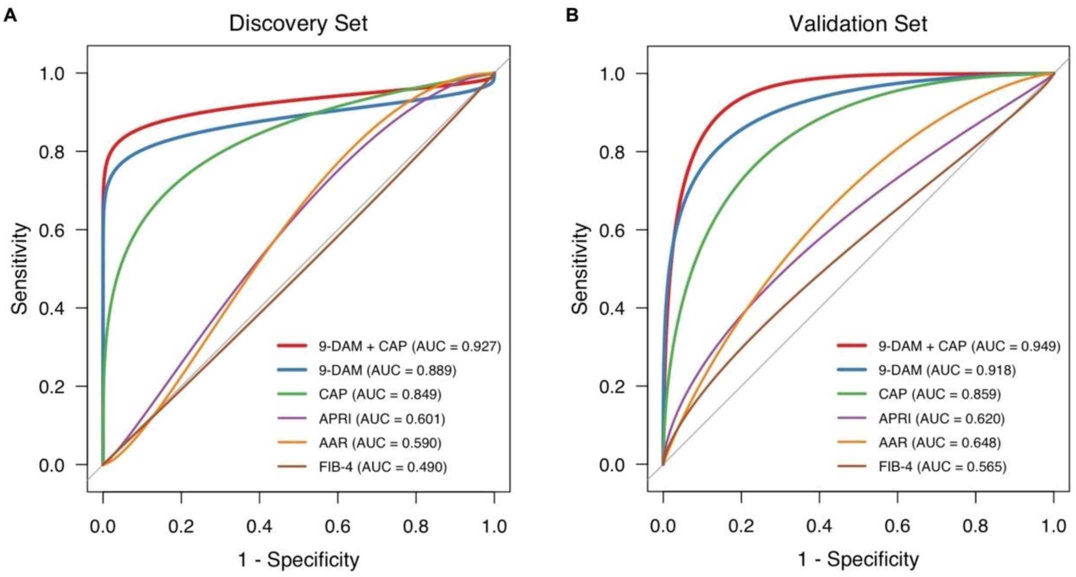

Fig. S2 Performance comparison between the 9-DAM panel and other non-invasive tests. (**A**-**B**) Receiver operating characteristic curves of the 9-DAM panel and clinical non-invasive tests in both the discovery and validation sets.

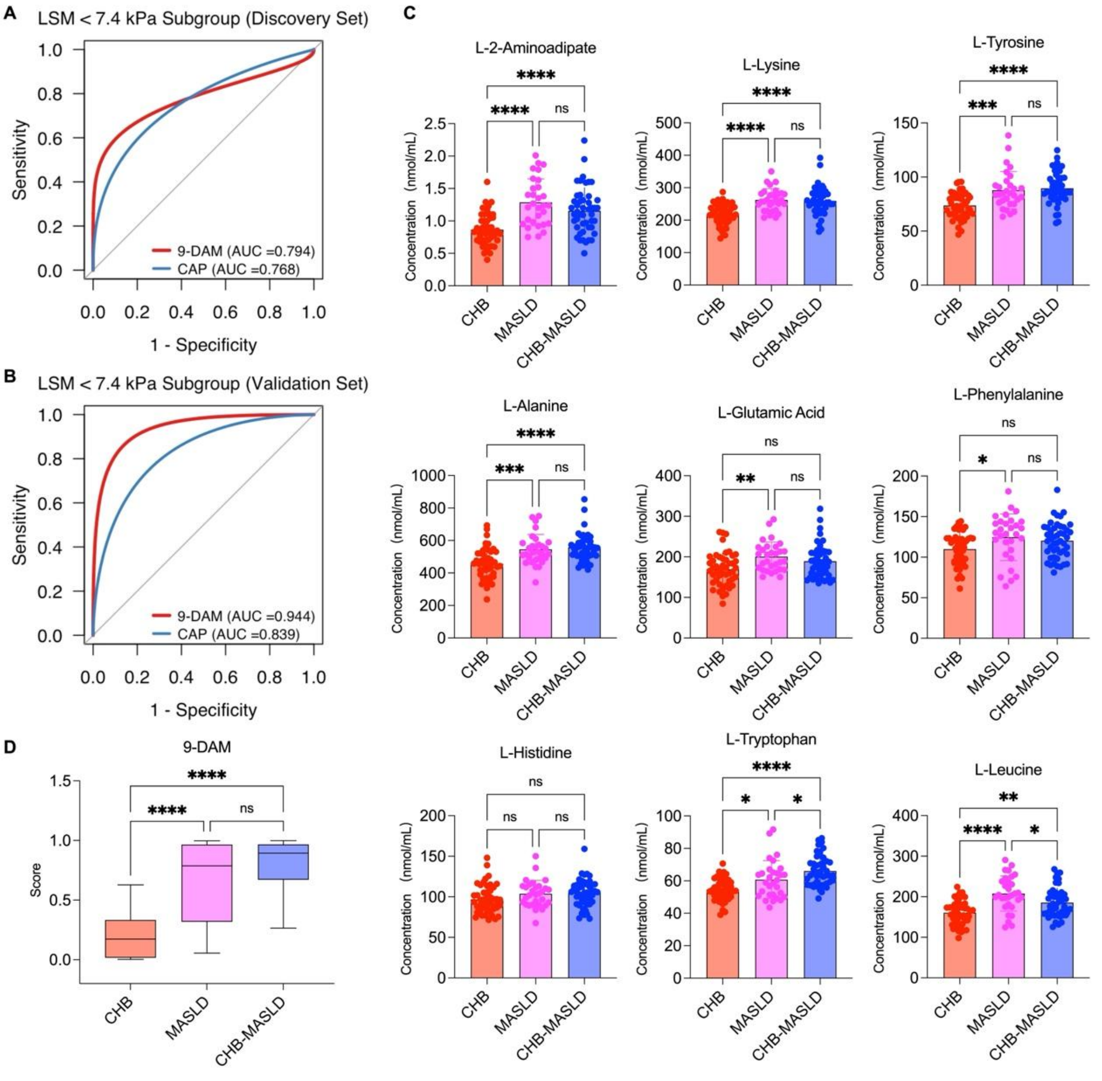

Fig. S3 Validation of the 9-DAM panel's specificity for hepatic steatosis. (**A-B**) ROC curves of the 9-DAM panel in the low-fibrosis subgroup (LSM < 7.4 kPa) within the discovery (**A**) and validation (**B**) sets. **(C)** Concentration comparisons of the nine amino acids across CHB, MASLD, and CHB-MASLD groups. **(D)** Comparison of the 9-DAM score among the CHB, MASLD, and CHB-MASLD groups. Data were analyzed using one-way ANOVA followed by Tukey's post hoc test for multiple comparisons and are presented as mean ± SD. *$p < 0.05$, **$p < 0.01$, ***$p < 0.001$, ****$p < 0.0001$.
